# Influence of Successive Wheelchair Tennis Matches on Handgrip Strength in High-Level Male Players

**DOI:** 10.3390/ijerph20064842

**Published:** 2023-03-09

**Authors:** Alejandro Sánchez-Pay, José Pino-Ortega, David Sanz-Rivas

**Affiliations:** 1 Human Performance and Sports Science Laboratory, Faculty of Sport Sciences, University of Murcia, 30720 San Javier, Spain; 2 Faculty of Sport Sciences, University of Murcia, 30720 San Javier, Spain; pepepinoortega@um.es; 3 Tennis Research Group, Department of Methodology and Professional Specialization in MEDAC, 28037 Madrid, Spain; dsanzrivas@gmail.com

**Keywords:** strength, racket sports, wheelchair, fatigue, handgrip

## Abstract

The purpose of this study was to examine the effects on upper strength in high-level male players playing four successive wheelchair tennis (WT) matches. Eight international WT players took part in a competition, playing one match per day over four consecutive tournament days. Before and after the match, the maximal isometric handgrip strength was measured on the dominant and non-dominant hand. Additionally, each player was equipped with one radiofrequency and IMU device on their wheelchair to control his activity profile (distance). The results showed significant differences between successive matches, with decreasing dominant handgrip strength (*p* = 0.02, η^2^ = 0.043), and there was a significant interaction between successive matches and the accumulated distance (*p* = 0.013, η^2^ = 0.049). The pre- and post-match strength values of the dominant hand decreased throughout the matches over a number of days, and post hoc analysis showed differences between the first and fourth matches only in pre-match strength (49.06 ± 6.96 vs. 45.94 ± 7.1; *p* = 0.045; ES: 1.04) but not in the non-dominant hand. Successive matches caused a decrease in the strength values of the WT players, mainly in the dominant hand. These results should be taken into account in the recovery and prevention of injuries in competitions with successive matches.

## 1. Introduction

In wheelchair tennis (WT) knockout tournaments, winning players continue to compete, while losers are eliminated. This means that some players must play successive matches (sometimes two per day) in tournaments lasting 4–5 days. Maintaining high levels of hand strength during the tournament is crucial for force transmission to the wheelchair rim, as well as the avoidance of injury; however, the influence of successive WT matches on hand strength has not yet been analysed.

WT matches are always played to the best of three tie-break sets, and the ball can bounce twice before it is hit [1]. A high-level WT match lasts 60–70 min [2,3,4,5], with rallies of 5–7 s [2,3] and three strokes per rally [2,3,6,7]. A WT tennis match is intermittent, multidirectional and non-random, which challenges the participant to change direction many times [5]. During the game, a player needs to perform specific movements, including starting, sprinting, braking and turning [8]. These specific movements are specifically related to propelling the wheelchair while holding a tennis racket [9].

The wheelchair propulsion technique is defined as the delivery of power to the hand rim of the wheelchair. Hand rim wheelchair propulsion is a complex bimanual motor task, during which the bimanually applied forces on the rims determine both the velocity and direction of locomotion [10]. There are multiple factors that seem to affect the wheelchair propulsion technique, and one of the most important is power output [11]. Moreover, propelling the wheelchair while holding a racket has a negative influence on the velocity of movement [9,12], which may be due to an ineffective push technique resulting in the low effectiveness of force application [9], as well as being a possible cause of injury [10]. Moreover, previous studies have shown a hand strength correlation of 0.58 with service speed [13]. In this sense, the fact that hand strength values may decrease throughout successive matches could compromise the physical performance of WT players and affect the mechanics of stroke production in terms of accuracy and power [14]; however, scientific investigations of this sport and this topic are scarce compared with other sports.

Previous studies on racket sports such as tennis or badminton have analysed the influence of matches on physical performance [15,16,17,18,19,20,21,22]. In general, researchers have used different tests to address this concern. Vertical jumping, sprinting, agility or isometric strength of the hips have been used to measure lower body strength [15,16,17,18,20,21], while isometric strength of the shoulder or hand has been used for the upper body [15,18,20,21,23]. However, findings from such studies, in addition to being contradictory, cannot be extrapolated to WT, since the players use the same limb to move and to perform a stroke, and this activity is permanent during a match, even during the rest time (where the player also has to propel the wheelchair). Therefore, the aim of this study was to analyse the effects of playing four successive WT matches on upper strength in high-level male players.

## 2. Materials and Methods

### 2.1. Participants

The best eight national-ranked male WT players (aged 38 ± 10) participated in sixteen singles WT matches. In total, 4 players were ranked among the international top 40 with Paralympic participation, and the other 4 were ranked among the international top 150 with no Paralympic participation. All of them played in the Open category. The characteristics of the participants are shown in Table 1. The study was conducted according to the Declaration of Helsinki of 2013 and approved by the Bioethics Commission of the local university (ID 2803-2020).

### 2.2. Procedure, Measures and Data Processing

The best eight players classified at the end of a season played a total of sixteen matches in the National Master Cup. In this competition, all the players play 4 matches on clay outdoor courts in a round-robin system with subsequent elimination. All matches used a type 2 ball, and the matches were played to the best of three sets with a 10-point super-tie-break in the third set, following the ITF regulations [1]. The players played one match per day between 10 a.m. and 12 a.m. so that they had a rest time of approximately 24 h.

**Figure 1:**
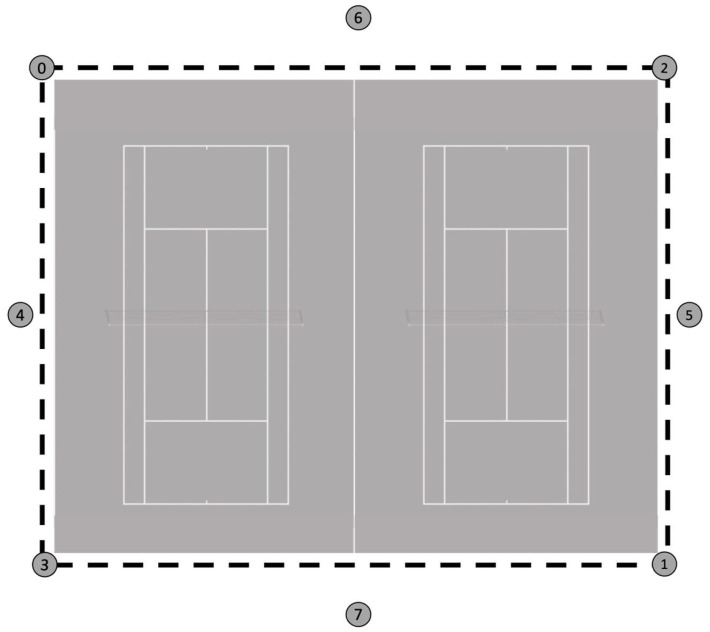
The ultra-wideband reference system used during the study.

*Activity profile (distance)*. The distance covered was measured using a radiofrequency through Local Positioning Systems (LPS) and inertial measurement units (IMU; WIMU PRO™, RealTrack Systems, Almeria, Spain). This system works similarly to the Global Position System (GPS), where the satellite network is replaced by a set of antennae placed around the field [24]. In this case, eight antennae were positioned around tennis courts, following previous studies on others racket sports [25]. The antennae were set up outside the perimeter of the tennis courts, positioned three metres high (Figure 1). The IMU device had its own internal microprocessor, 2 GB flash memory, and a high-speed USB interface to record, store and upload data. The devices were powered by an internal battery with 4 h of life, weighed 70 g in total, and measured 81 × 45 × 16 mm. Each device contained, among other sensors, a 10 Hz GPS and 33 Hz UWB. Each tennis player was equipped with one IMU device positioned within the frame of the wheelchair under the seat. This system has previously been used in other studies reporting high values of reliability and validity [24]. Data collection commenced at the beginning of warm-up on the tennis court and finished at the end of each match. In this way, the total external load (distance covered) was recorded and was paused during stoppages.

*Grip Strength*. The maximal handgrip strength was measured with a portable hand dynamometer, Smedley III T-18A (Takei, Tokyo, Japan). The hand dynamometer has a range between 0 and 100 kg with 0.5 kg increments and an accuracy of ±2 kg. The handgrip strength test was performed in the wheelchair sitting position, with the elbow extended and the arm positioned with the dynamometer parallel to the subject’s side and not touching the wheelchair [26,27]. Participants were asked to perform a maximal voluntary contraction, squeezing the dynamometer as hard as possible, for 3 s [28]. The rest time between each attempt was 2 min. The maximum force (kilograms) achieved (with 2 trials for both hands) was recorded. To avoid interfering with the normal course of the competition, the measurements were taken inside the tennis court. The test was performed on both the dominant and non-dominant hands. The test was performed before specific warm-ups on court and immediately after finishing the match.

### 2.3. Statistical Analysis

Firstly, a descriptive exploration of the data obtained was carried out and the mean and standard deviation were calculated. The Shapiro–Wilk test confirmed that the data were normally distributed. The Levene test confirmed that the variances in the data were homogenous. The repeated measures analysis of variance (repeated measures ANOVA) was performed to a) compare the distance in the four matches and b) compare the handgrip strength between the four matches, and accumulated distance was included as a covariable. An eta-squared effect size of η^2^ = 0.02 was considered as a small effect size, an effect size of η^2^ = 0.13 was considered as a medium effect size, and η^2^ = 0.26 was considered as a large effect size. Holm’s correction was used for the post hoc tests to assess cases where differences occurred. The effect size (ES; Cohen’s d) was included and evaluated as follows: 0–0.2 = trivial; 0.2∓0.5 = small; 0.5∓0.8 = moderate; and >0.8 high. A significance level of *p* < 0.05 was established. Statistical analyses were performed using IBM SPSS v. 25.0 (IBM Corp., Armonk, NY, USA) and JASP v 0.14 (computer software, Amsterdam, The Netherlands).

## 3. Results

Table 2 shows the results of the hand strength (dominant and non-dominant) tests before and after each of the four matches. The percentage variation between the pre- and post-match values, as well as that between the dominant and non-dominant hands by match, is also shown.

**Figure 2:**
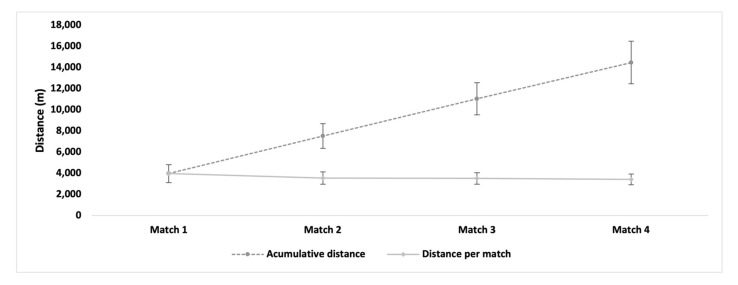
Accumulated distance per match and throughout successive matches. Lines denote the standard deviation.

Figure 2 shows the distance covered per match, as well as the total accumulated distance during the tournament. Players covered 3622 ± 832 m per match. No statistically significant differences were found in the distance covered per match (*p* > 0.05). The players accumulated a total distance of 14,490 ± 2005 m during the tournament.

**Figure 3:**
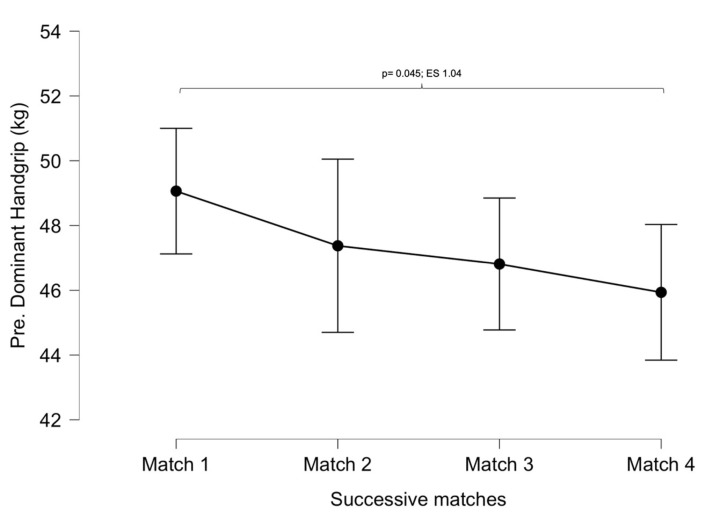
Pre-match dominant handgrip strength throughout successive matches. Lines denote the mean (95 CI).

The results for dominant handgrip strength pre-match are shown in Figure 3. For dominant handgrip strength, significant differences were identified between successive matches (F_(3, 18)_ = 4.214, *p* = 0.02, η^2^ = 0.043). There was a significant interaction of successive matches and accumulated distance (F_(3, 18)_ = 4.787, *p* = 0.013, η^2^ = 0.049). The pre-match strength values in the dominant hand decreased throughout the matches, and the post hoc analysis showed differences between the first and fourth match (*p* = 0.045; ES: 1.04).

**Figure 4:**
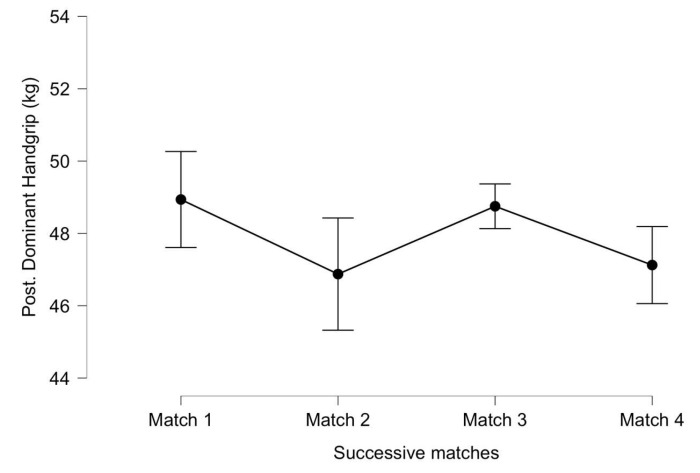
Post-match dominant handgrip strength throughout successive matches. Lines denote the mean (95 CI).

For the post-match dominant handgrip strength (Figure 4), significant differences were not identified between successive matches (F_(3, 18)_ = 1.270, *p* = 0.310, η^2^ = 0.006). There was no significant interaction between successive matches and the accumulated distance (F_(3, 18)_ = 1.171, *p* = 0.332, η^2^ = 0.006), although a high ES was found between the first and second matches (*p* = 0.054, ES = 1.03) and also between first and fourth matches (*p* = 0.08, ES = 0.91).

**Figure 5:**
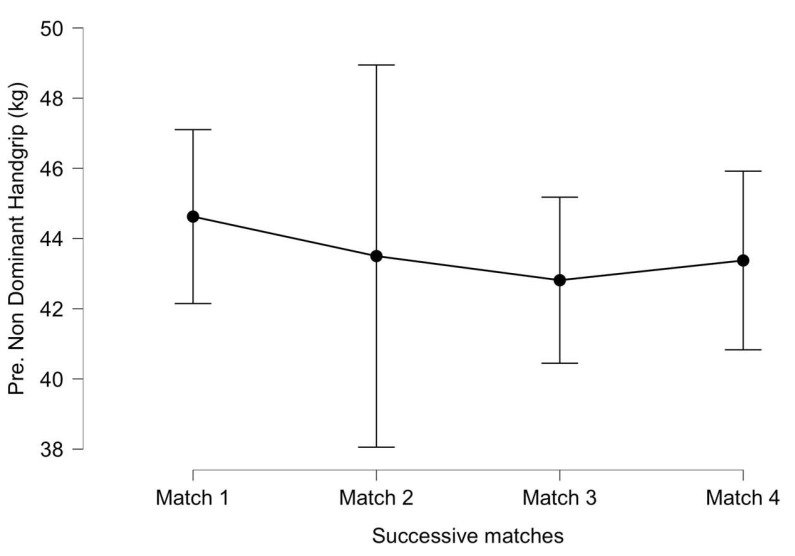
Pre-match non-dominant handgrip strength throughout successive matches. Lines denote the mean (95 CI).

For the pre-match non-dominant handgrip strength (Figure 5), significant differences were not identified between successive matches (F_(3, 18)_ = 0.134, *p* = 0.939, η^2^ = 0.001), nor was a significant interaction between successive matches and the accumulated distance (F_(3, 18)_ = 0.172, *p* = 0.914, η^2^ = 0.001) identified. The effect sizes were small between first and third matches (ES = 0.29) and between the first and fourth matches (ES = 0.20).

**Figure 6:**
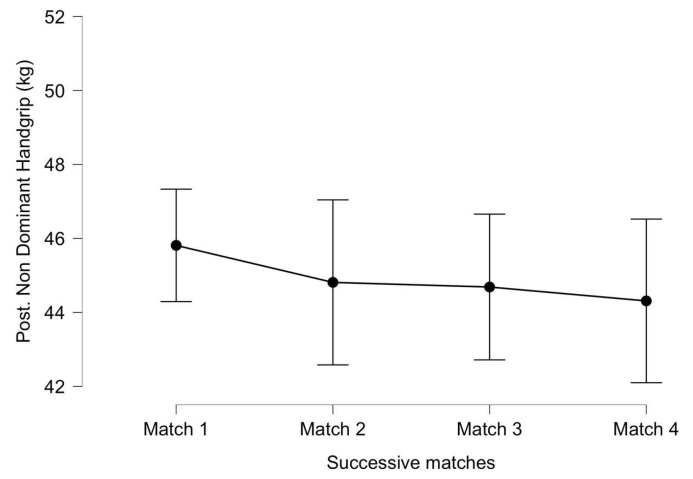
Post-match non-dominant handgrip strength throughout successive matches. Lines denote the mean (95 CI).

The results for the post-match non-dominant handgrip strength are shown in Figure 6. For the non-dominant handgrip strength, significant differences were not identified between successive matches (F_(3, 18)_ = 1.134, *p* = 0.362, η^2^ = 0.001). There was no significant interaction between successive matches and the accumulated distance (F_(3, 18)_ = 1.077, *p* = 0.384, η^2^ = 0.0001). The post-match strength values of the non-dominant hand seemed to decrease throughout the matches, although the post hoc analysis showed a small effect size between the first and third matches (ES = 0.33) and between the first and fourth matches (ES = 0.44).

## 4. Discussion

The aim of this study was to analyse the effects of playing four successive WT matches on upper strength in high-level players in relation to the accumulated distance over a number of days. To the best of our knowledge, this is the first study to analyse the effects of successive WT matches on hand strength. The main finding was that the dominant hand strength pre-match values decreased throughout the matches. These results should be taken into account when developing specific recovery programs for accumulated fatigue in the upper body.

In the initial grip test (pre-match 1), the players showed values of 49.1 kg in the dominant hand and 44.6 kg in the non-dominant hand (Table 2). These values are similar to the values reported for wheelchair basketball, being near to 45 kg [27,29], and for racket sports such as tennis (50 kg) [28] or badminton (44–47 kg) [18,30]. The influence of the match on the handgrip strength has been studied in other racket sports, such as badminton, with different results [18,20,30]. In the study of Abián-Vicent et al. [20,30], measurements were conducted in official competitions with a duration between 34 and 42 min, and no decrease in hand strength was observed. On the contrary, in the study by Phomsoupha et al. (2019) [18], a decrease in hand strength was found after 60 min of simulated matches. The differences in the results found between both studies could be due to the playing time; in Phomsoupha et al.’s (2019) study, the strength values decreased after 50 min. This suggests that players are able to maintain a high level of isometric strength for the duration of a classic match lasting less than 35 min [18]. A typical duration of a high-level WT match is 60–70 min [2,3,4,5] longer than that of a badminton match, resulting in a probable decrease in hand strength in players in WT matches of a typical duration. The pre-match strength values in the dominant hand decreased throughout the matches, showing differences between the first and fourth matches (*p* = 0.045; ES: 1.04). The hand strength values decreased by 4.6% between the first and third matches and 6.4% between the first and fourth matches. Keeping high levels of hand strength is important for several reasons. On the one hand, forces applied on the rims determine both the velocity and direction of locomotion [9], and the low effectiveness of force application could result in an ineffective push technique, as well as acting as a possible cause of injury [10]. On the other hand, previous studies have shown a grip strength correlation of 0.58 with service speed [13]. The serve is one of the most important strokes in the modern game [31], and an increasing serve speed reduces the time required for the opponent to successfully return the ball and increases the probability of the server’s dominance in the rally or of winning a direct point [32]. In addition, previous studies have shown a significant reduction in service speed (−1.8 m·s^−1^) after prolonged matches (3 h) [19]. Although the present study did not record service speeds, it can be assumed that there may be a negative influence on the hitting speed due to a decrease in dominant hand strength.

Overall, the players covered an average distance of 3622 m per match (Figure 2). These values are similar to those found in previous studies, ranging between 3000 and 4500 m per match on hard courts [6,33,34]. The results of this study show that tennis players covered similar distances over 4 consecutive days, and no significant differences were found between matches (Figure 2). Previous studies on tennis showed that players reduced their external physical loads through decreased movement over 4 consecutive days of prolonged competitive tennis, indicating a change in the external load in response to a tactical modification, given that the rating of perceived exertion was stable, and the effective playing time was slightly lower in matches 3 and 4 [21]. In our study, the players maintained a similar covered distance throughout the tournament, indicating that the succession of matches does not affect the external load in terms of distance.

Muscle fatigue is defined as a reduction in the maximum capacity of the muscle to generate force [35]. Some previous studies on racket sports have shown a relationship between the reduction in maximal force or neuromuscular performance and an increase in serum creatine kinase, an indirect method for detecting muscle injury [16,17,36]. It seems that muscle damage is one factor behind the impairment of physical performance during a tournament [16]. Recovery times are important for ensuring that players can maintain a high level of performance throughout successive matches. Thus, a recovery time of 48 h seems to be sufficient to recover service speed values [16]; however, in WT tournaments, the matches are held every 24 h or less, meaning that these recovery times are not possible for disabled players. This knowledge is very important for strength and conditioning trainers, enabling them to implement specific training sessions during the week while modulating the training loads. This can optimize the training performance and, consequently, reduce the injury risk.

Despite being the first study to analyse the influence of successive matches on hand strength during an official tournament among high-level wheelchair players, there are several limitations that should be considered. First of all, the sample size is somewhat small, which rendered it impossible to use more precise statistical methods. However, the eight best international wheelchair tennis players from the same country participated in the study, half of whom were Paralympic players, and it is always a difficult task to recruit high-level players. Additionally, the analysis concerns only handgrip strength and physical activity (distance). Furthermore, further research should include other metrics, such as accelerations and decelerations, as well as internal and external shoulder strength, which is important both for the tennis strokes and for biomechanical movements in the wheelchair. In addition, due to the nature of the competition (an official tournament) and the level of some players (professionals and Paralympic players), the individual recovery strategies could not be controlled, which could have affected the results.

## 5. Conclusions

In summary, successive matches over a number of days cause a decrease in the pre-match strength values of wheelchair tennis players, mainly in the dominant hand. The decrease in strength values is more pronounced before the match, which suggests that 24 h of recovery is not enough to achieve the initial levels, and fatigue increases over successive matches during the tournament. In addition, practitioners working in wheelchair tennis have an important area for future research in efforts to alleviate fatigue and accelerate recovery. From a practical point of view, it seems to be important to include post-match recovery modalities immediately after matches (e.g., cold water therapy), which could be an effective method used to alleviate fatigue and accelerate recovery [36].

## Figures and Tables

**Table 1:** Characteristics of the sample of wheelchair tennis players participating in the study.

N	NR	IR	Age	Weight (kg)	Training (h)	Experience	Disability
1	1	Top 15 *	20	65	15	10	At
2	2	Top 15 *	26	61	20	14	At
3	3	Top 40 *	42	63	8	22	SCI D12
4	4	Top 40 *	36	67	8	7	SCI L12
5	5	Top 60	37	72	10	5	SCI D11
6	6	Top 100	46	71	6	6	Af
7	7	Top 100	41	70	6	12	Af
8	8	Top 150	47	61	6	8	SCI L3

NR: national WT ranking. IR: international WT ranking. Training (h): playing training hours per week. Experience: WT playing experience (years). * Paralympic players. At: amputation tibial height. Af: amputation femoral height. SCI: spinal cord injury. D: dorsal. L: lumbar.

**Table 2:** Descriptive analysis of handgrip strength of dominant and non-dominant hand in successive WT matches.

Variable	Match 1		Dif	Match 2		Dif	Match 3		Dif	Match 4		Dif
	Pre	Post	%	Pre	Post	%	Pre	Post	%	Pre	Post	%
Dominant	49.1 (7.0)	48.9 (6.5)	−0.3	47.4 (6.4)	46.9 (7.4)	−1.0	46.8 (6.3)	48.8 (6.9)	4.1	45.9 (7.1)	47.1 (5.6)	2.6
Non-dominant	44.6 (7.8)	45.8 (9.2)	2.7	43.5 (8.9)	44.8 (9.0)	3.0	42.8 (8.6)	44.7 (9.0)	4.4	43.4 (8.8)	44.3 (8.4)	2.2
Dif. Bilateral (%)	9.0	3.4	−5.6	8.2	4.4	−3.8	8.5	8.3	−0.2	5.6	6.0	0.4

Pre- and post-match values expressed as kg. Dif = Difference.

## Data Availability

The data presented in this study are available on request from the corresponding author.
